# A Scoping Review of Sleep Apnea: Where Do We Stand?

**DOI:** 10.3390/life13020387

**Published:** 2023-01-31

**Authors:** Rahim Hirani, Abbas Smiley

**Affiliations:** 1 School of Medicine, New York Medical College, Valhalla, NY 10595, USA; 2 Department of Surgery, Westchester Medical Center, New York, NY 10595, USA

**Keywords:** sleep apnea, morbidity, upper airway, risk factors, pathogenesis, CPAP

## Abstract

Obstructive sleep apnea (OSA), a condition in which there is a recurrent collapse of the upper airway while sleeping, is a widespread disease affecting 5% to 10% people worldwide. Despite several advances in the treatment modalities for OSA, morbidity and mortality remain a concern. Common symptoms include loud snoring, gasping for air during sleep, morning headache, insomnia, hypersomnia, attention deficits, and irritability. Obese individuals, male gender, older age (65+), family history, smoking, and alcohol consumption are well recognized risk factors of OSA. This condition holds the ability to increase inflammatory cytokines, cause metabolic dysfunction, and increase the sympathetic output, all of which exacerbate OSA due to their effect on the cardiovascular system. In this review, we discuss its brief history, risk factors, complications, treatment modalities, and the role of clinicians in curbing its risk.

## 1. Introduction

Obstructive sleep apnea (OSA) is an illness of the upper airway that causes intermittent cessation in ventilation, causing hypoxia and hypercapnia due to the periodic collapse of the trachea [1,2]. It is estimated that around 936 million adults aged 30–69 years, both men and women, suffer from mild to severe OSA, and approximately 425 million adults aged 30–69 years have moderate to severe OSA globally [3], making it a worldwide concern for healthcare providers and health delivery systems. Along with a relatively higher incidence, it is particularly concerning given that a high number of patients remain undiagnosed, resulting in an unpredictable epidemiology of this disease [4].

Although various diagnostic and therapeutic advances have been made over the decades to better manage this illness, morbidity and mortality rates remain high; OSA is reportedly associated with a 1.9-times-higher risk in all-cause mortality and 2.65-times-higher risk of mortality related to cardiovascular issues [5,6]. Along with cardiovascular issues, OSA has been shown to be independently associated with stroke, cancer, and many other illnesses [6,7]. Therefore, further studies are required to better understand the associated risk factors and devise novel treatment modalities.

The economic burden of undiagnosed sleep apnea in the US is approximately USD 150 billion, and experts suggest an annual saving of around USD 100 billion if we are able to diagnose and treat every patient in the US who has OSA [8]. Therefore, along with the advances in the management of this illness, it is also important to investigate the causes and solutions in order to increase the diagnosis rate and risk factors associated with OSA.

In this article, we review the etiology and various established risk factors associated with OSA. Additionally, we review the evidence and discuss various treatment modalities used to better manage patients with OSA. Lastly, we review some complications and the role of healthcare providers to reduce associated mortality and morbidity rates.

## 2. Sleep Apnea: A Brief History and Risk Factors

Although the official naming and discovery of sleep apnea reportedly occurred in the 1960s, it is by no means a new disorder [9]. While it is true that it is only receiving relatively more attention due to further advances in diagnosing and managing the disease, the symptoms first appeared approximately 2000 years ago and were lumped together using the term “Pickwickian syndrome” in the 19th century [10,11]. For years, the initial focus was on the process of understanding the intermittent closure of the upper airway; however, the late 1960s brought a fresh perspective to observe the various symptoms and risk factors at the same time, though limited by methodological difficulties at the time [9,12]. Research studies were mainly conducted via observing dogs and treating the condition with tracheotomies [13]. Though an earlier concept of continuous positive airway pressure (CPAP) using a customized mask in the 1970 and 1980s further advanced modern management [14]. To date, polysomnography, including electrocardiogram, sleep staging, electromyogram, and electroencephalogram, is the gold standard for diagnosing OSA, while home sleep apnea testing (HSAT) is an alternative method with some limitations [15,16].

There are four reported endotypes of OSA: loop gain, upper-airway collapsibility, arousal threshold, and upper-airway dilator muscle response, which is also knows as compensation [17]. Loop gain is basically the ventilatory response-to-disturbance ratio estimated by ventilation characteristics during obstructed breathing episodes [18]. It is usually noted as a drop in CPAP, suggesting a decrease in ventilation as compared to the holding pressure. This drop in ventilation leads to the accumulation of CO2, and thus, an increase in ventilatory drive. This increase can be estimated by measuring the ventilatory overshoot from the holding pressure of CPAP, providing a ratio for response and disturbance in ventilation [17,19,20]. Upper-airway collapsibility measures the propensity for collapse as reported in patients among OSA. Although there are several techniques to measure this mechanistic variable, negative pressure pulses seem to provide a reliable estimate as it is rapid and thus less likely to be influenced by external behaviors [21]. Arousal threshold is essentially the compensatory drive of ventilation that produces arousal [18]. This variable is a measure of the propensity to wake up from sleep given the changes in negative intra-thoracic pressure [18,20,22]. Lastly, patients with ineffective upper-airway dilator muscle endotypes have a decreased tone of dilator muscles, particularly genioglossus, the largest extrinsic muscle of the tongue [23]. The absence or presence of these endotypes can manifest differently in individuals that can subsequently have an impact on the severity of the disease. These individual endotypes can also be targeted individually, or in combination, using various techniques that are briefly discussed later in this review article.

**Figure 1:**
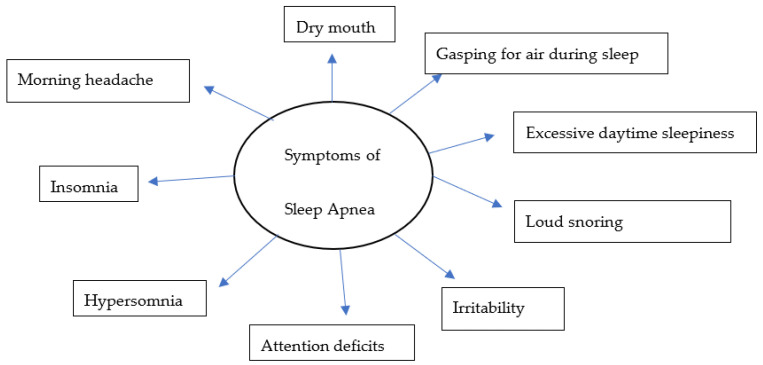
A model for common symptoms of sleep apnea.

**Figure 2:**
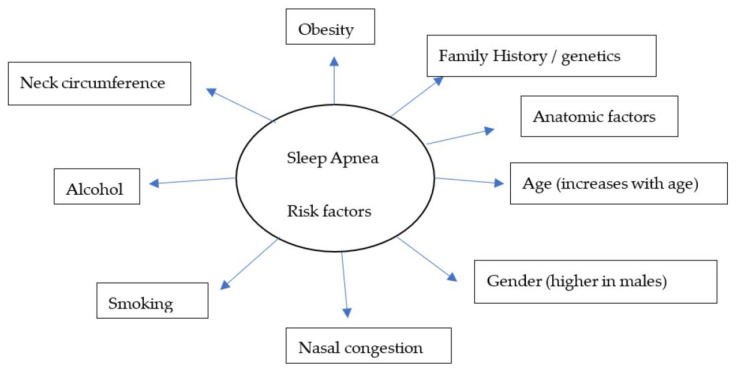
A model for widely recognized risk factors of sleep apnea.

Over the years, there have been several advances, including the identification of symptoms (Figure 1) and various risk factors, to better diagnose and manage individual conditions. Loud snoring, gasping for air during sleep, xerostomia, insomnia, hypersomnia, nocturnal choking, and attention deficits are some of the many symptoms that can be observed in patients with OSA [24,25,26,27]. Some of the most important risk factors of sleep apnea are discussed in this review (Figure 2).

### 2.1. Obesity

Obesity has been identified as one of the main components contributing to OSA [28,29]. Many correlations have been established between weight, BMI, waist-to-hip ratio, neck circumference, and severity of OSA. The sleep heart healthy study was one of the landmark studies that established the same ideas; the study showed an increase in the apnea-hypopnea index (AHI) by approximately five-fold in men and two-fold in women over the course of their study [30]. Peppard et al. in their population-based prospective cohort study of 690 randomly selected Wisconsin residents also demonstrated a six-fold increase in the odds of developing OSA with a mere 10% increase in weight, while weight loss resulted in decreasing severity among patients [31], suggesting a reciprocal relationship between these two variables.

### 2.2. Family History/Genetics

Several studies have reported some underlying causes of OSA to have a genetic component, suggesting its hereditary nature. However, these results should be carefully evaluated as OSA as a disorder is a complex interplay between genetics and environmental factors. The Cleveland Study was another landmark study in investigating OSA and its link to genetics. This genetic–epidemiologic study concluded that OSA is more prevalent in relatives of index probands of OSA as compared to their control counterparts [32]. Additionally, Ferini-Strambi et al. in their comparative study showed a higher prevalence of snoring among monozygotic twins, which is one of the primary symptoms of OSA [33]. Other biomarkers via genome-wide linkage studies have also been investigated to establish an association between OSA and various genes. One study showed such an association between a polymorphism in the angiopoietin-2 gene (ANGPT2) and mean nocturnal oxygen saturation, which is a commonly used marker to determine severity in OSA [34]. Furthermore, similar studies have established polymorphisms in tumor necrosis factor-a (TNF-a), prostaglandin E2 receptor EP3 subtype (PTGER3), and Lysophosphatidic acid receptor 1 (LPAR1) to be a risk factor associated with OSA [35,36]. Therefore, further studies and the biological significance of these polymorphisms in conjunction with OSA are warranted.

### 2.3. Age and Gender

Generally, OSA has been shown to be more prevalent in men with a two-fold-greater likelihood in people older than 65 years as compared to middle-aged adults aged 30–50 years [37]. Additionally, the prevalence was shown to be around 5% in middle-aged females and 12% in their male counterparts [38]. These estimates are around 12–32% in patients aged 65 years or older [37,39]. However, the severity of the disease varies among elderly individuals and could even be milder than the severity observed in adults [40]. In the elderly, the disease is said to be manifested differently, resembling behavioral and cognitive impairments mimicking dementia [41,42].

According to the current literature, there is a higher prevalence of OSA among men compared to women [43,44,45,46]. In a recent study of 1208 people between 20 and 81 years of age with 46% of the cohort being female, an estimated prevalence of OSA was 33% among women when AHI was more than equal to 5%, while it was 59% among men [44]. Even with an AHI of more than or equal to 15%, the prevalence was higher in men when compared to women; 30% vs. 13%, respectively [44]. This effect was not only restricted to prevalence; OSA has been reported to be more severe in men with more specific symptoms suggestive of OSA when compared to women. While men frequently report snoring, gasping, attention deficits, insomnia, snorting, and apnea, women are reportedly presented with more non-specific symptoms, such as headache, fatigue, depression, and anxiety [43]. The presence of only non-specific symptoms then could make it challenging for a physician to perform a correct diagnosis. This also helps explain the results that women are diagnosed at advanced ages and with a higher BMI as compared to men. The difference in gender, generally speaking, could also be due to a differing body-fat distribution in males vs. females, with males having more adipose tissue in the neck region, resulting in a higher susceptibility to airway collapse [47,48]. Although the pharyngeal cross-sectional area is reported to be similar among men and women, men are noted to exhibit greater upper-airway collapsibility. This could be accounted for by the presence of a longer airway length and larger volume of soft tissues on the lateral pharyngeal walls in men [43,49]. Hormonal differences are yet another factor that plays a role in the differing prevalence of OSA among men and women. Previous studies have shown how ventilatory response is affected and AHI is increased in hypogonadal men with an acute administration of testosterone [43,50]. In one study of testosterone replacement therapy among hypogonadal men, Matsumoto et al. reported not only a significant decrease in the ventilatory drive in patients receiving testosterone, but also noted the new induction of OSA, and an exacerbation of symptoms in patients previously diagnosed with OSA [50]. Years later, after this study, it was deciphered that an acute administration of testosterone enhances the ventilatory instability and the loop-gain of the ventilatory system as a consequence of an increase in the ventilatory response to hypoxia [19], increasing the predisposition to OSA in men.

OSA among pregnant and post-menopausal women is one area where there is a lack of research. While it has been reported that sleep-disordered breathing is more severe in postmenopausal when compared to premenopausal women [51], it is unclear whether a decreased production of female hormones plays a role in this exacerbation. Moreover, symptoms of OSA can also be more difficult to identify or interpreted as menopausal manifestations, leading to misdiagnosis [45]. Therefore, these gender differences could mostly be explained by anatomic and physiologic variabilities, a difficulty identifying and categorizing non-specific symptoms, and underdiagnosis due to physician biases [48].

### 2.4. Smoking and Alcohol

Several studies have cited smoking and alcohol as risk factors for OSA. This could be explained by a general decrease in sleep latency, difficulty in initiating sleep, and irregular sleeping patterns after smoking or drinking [52]. The chemicals consumed during smoking can also result in local inflammation and fluid retention in the upper airway, which could exacerbate these symptoms. Where many studies have observed a positive correlation between smoking and OSA, such as in a study by Kashyap et al., who reported the occurrence of OSA to be approximately twice as likely in current smokers as compared to previous smokers and non-smokers combined [53], many other studies have shown the opposite association, or did not observe smoking to be an independent variable for OSA. However, the number of cigarettes consumed per day was still reported to be higher among more severe forms of OSA [54]. Some studies have also reported smoking addiction due to untreated OSA [55]. This variability in results suggests an inconclusive consensus about the role of smoking in OSA progression and severity; thus, further studies are required to elucidate the relevant mechanisms involved.

On the other hand, the role of alcohol seems to be relatively established among OSA patients. The studies show a general consensus that alcohol is positively correlated with an increased risk and severity of OSA by 25% [56]. The likely mechanisms include the relaxation of muscles in the neck and throat leading to airway collapse, decreased ventilatory responses to an increase in higher partial pressure of CO2 and lower pressure of oxygen, and reduction in muscle activity in the tongue [48]. While we know that alcohol can increase the risk of OSA, it would be beneficial to understand if these effects are impacted by individual race, metabolic and immune status, number of drinks consumed per week, and whether individuals are suffering from any other comorbidities. Answers to these questions will allow for a better public health policy.

### 2.5. Inflammation

Inflammation plays an integral role in the induction, progression, and exacerbation of OSA. Over the years, several inflammatory mediators have been corelated with the pathogenesis of OSA; however, some are more extensively researched and notably reported, including CRP, IL-6, IL-8, IL-33 and its receptor ST2, Pentraxin-3 (PTX-3), procalcitonin (ProCT), and TNF-a [57,58]. These mediators can play an important role as a biomarker to decipher the severity of OSA among patients. Particularly, PTX-3 as a predictor of OSA severity has garnered attention due to its consistent specificity and sensitivity across studies. For example, Sozer et al. reported a specificity and sensitivity of 91.7% for PTX-3 as a predictor of OSA among other inflammatory mediators [57]. They also reported a positive correlation between PTX-3 and BMI, suggesting a potential link between these two variables in the subsequent progression of the disease. Other studies have noted the importance of morning levels of PTX-3 as a sensitive biomarker, as patients with OSA could have a higher hypoxic state during sleep [59]. PTX-3 is essentially from the same family as CRP, an acute phase protein with a role in innate immunity, the regulation of inflammatory reactions, and apoptosis. Its role in the pathogenesis of OSA was further elucidated after treating patients with CPAP. In a study by Kobukai et al., there was a marked reduction in the morning levels of PTX-3 and CRP; however, only PTX-3 levels were shown to be significantly correlated with the severity of OSA using AHI [59].

CRP is another important biomarker that has been extensively researched in the pathogenesis of OSA. However, its association and specificity have been questioned in the last two decades, given the variable results across studies [58,60,61,62]. A strong relationship between OSA and obesity was established in earlier studies, and perhaps this relationship could distort the data if the patients were not optimally matched for BMI [63].

These inflammatory mediators are subsequent culprits in cardiovascular complications, metabolic dysfunction, and atherosclerosis [58], which is briefly discussed later in this article.

## 3. Complications of Obstructive Sleep Apnea

While OSA is a major concern on its own for any patient and their healthcare providers, it involves several other complications and sequelae that follows due to OSA pathogenesis and symptomatology. These complications often exacerbate their overall health and further its morbidity and mortality rates. Here, we discuss some of the most pressing complications that are caused by OSA.

### 3.1. Cardiovascular Diseases

**Figure 3:**
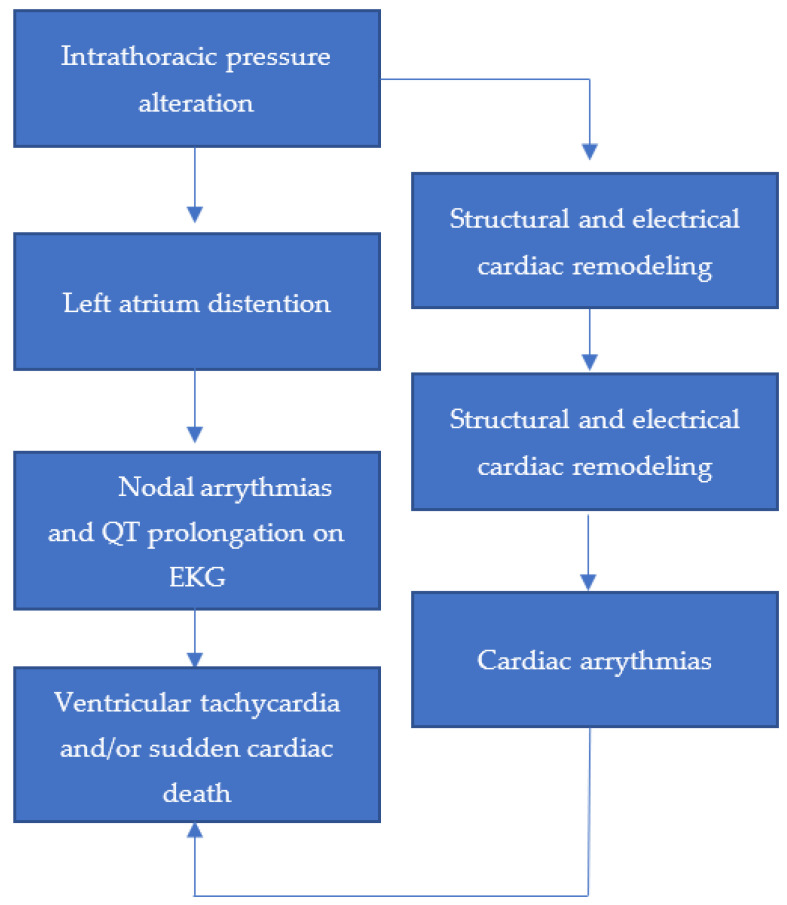
A mechanistic model for the impact of intrathoracic pressure alterations due to OSA on cardiac dysfunction.

Several studies have established a clear association between OSA and various cardiovascular diseases (CVDs), such as hypertension, stroke, coronary artery disease, and atrial fibrillation [64]. While there is no consensus on a well-adopted mechanism for this association, it is reported to include a heightened sympathetic activation, and in turn release of stress hormones, due to difficulty of breathing [65]. This effect is mediated by the hypothalamic–pituitary–adrenal axis, the activation of which has been shown to be corrected with the use of CPAP, along with a decrease in cortisol levels [65,66]. We know that hypertension is a major risk factor for CVD [67], which is prevalent among OSA patients when oxygen levels are decreased due to the narrowing of the airway. This hypertension that exacerbates the activation of the sympathetic system even causes coronary artery disease. OSA can also cause an abnormal heart rhythm, which is difficult to manage if there are other underlying heart conditions or comorbidities directly, or indirectly, affecting cardiovascular health [68]. It is estimated that approximately 30–50% of OSA patients are also diagnosed with cardiac arrhythmias, including atrial and ventricular premature extrasystoles, ventricular tachycardia, sinus arrest, and atrioventricular conduction block [69]. These acute triggers can be explained by previously studied arrhythmogenic mechanisms, where an alteration in the intrathoracic pressure leads to a stretch in the muscle of the left atrium, which causes distention. This distention in turn gives rise to increased atrial premature beats and QT interval prolongation on an electrocardiogram (EKG) [70]. As a result, ventricular tachycardia and/or sudden cardiac death due to this acute episode can ensue. The triggered activity and automaticity due to enhanced sympathetic discharge, and parasympathetic surge on the other hand during apnea, can cause sinus nodal disease and atrial fibrillation (Figure 3) [70,71]. It is important to note that while there is a strong association between OSA and CVD, many trials have failed to establish that these symptoms improve when treating for OSA. Therefore, further studies are required to understand the underlying mechanisms for better targeted therapies.

Other mechanistic intermediates include a change in intrathoracic pressure, platelet activation due to endothelial damage, and an increase in the levels of inflammatory cytokines, which could all have an impact on CVD [72]. The cytokines involved in these heightened inflammatory reactions are reported to be TNFa, IL-6, IL-8, and C-reactive protein (CRP), which are also associated with excessive daytime sleepiness among OSA patients, along with their action on endothelial damage and myocyte dysfunction [73]. Myocyte hypertrophy due to hypertension can also cause the remodeling of cardiac myocyte, resulting in fibrosis [70]. A similar result could be observed in OSA patients with comorbid obesity, which could enhance the renin–angiotensin–aldosterone system (RAAS) due to sympathetic activation. This in turn can also cause arrhythmias and sudden cardiac death.

### 3.2. Metabolic Dysfunctions

OSA has been linked to cause, and also manifest, several metabolic derangements, such as insulin resistance, type II diabetes mellitus, metabolic syndrome, and non-alcohol fatty liver disease [45,74]. In a nationwide study of 1,704,905 patients with OSA and an approximately equal number of controls, Mokhlesi et al. attributed a higher prevalence of type II diabetes and ischemic heart disease in men, while hypertension and depression were more prevalent in women as compared to their matched controls [75]. This prevalence of insulin resistance and glucose intolerance among OSA patients was estimated to be anywhere from 20% to approximately 70% [76]. It is important to note that these results are independent of obesity, which could be corroborated as a potential confounding factor. Additionally, while CPAP has been shown to be beneficial in the management of OSA, the results are inconclusive whether it helps in curbing the risks that come with dysfunctional glucose metabolism [76]. This does not only pose a concern for patients with OSA, but also for the healthcare system by further increasing the economic burden of diabetes [77].

**Figure 4:**
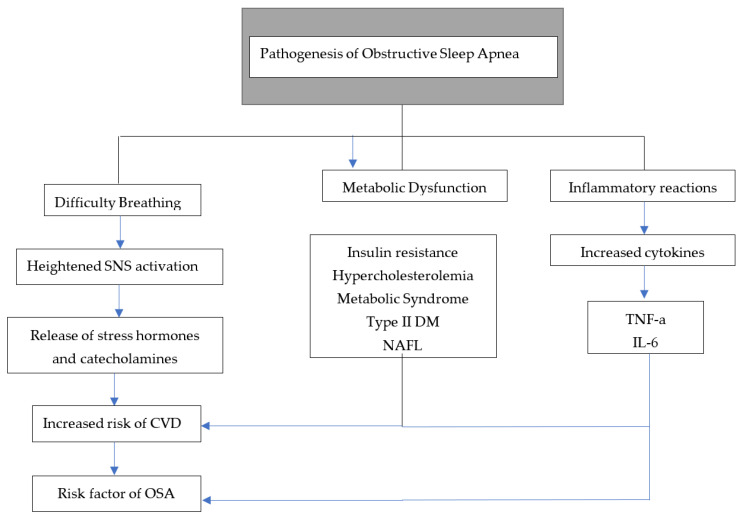
A mechanistic model for pathogenesis of OSA. Abbreviations: SNS = sympathetic nervous system, CVDs: cardiovascular diseases, OSA = obstructive sleep apnea, DM = diabetes mellitus, NAFL = non-alcohol fatty liver, TNF-a = tumor necrosis factor alpha, and IL-6 = interleukin 6.

As mentioned, not only OSA can cause diabetes, but diabetes could result in OSA as well. This bidirectional association could be explained due to some control of respiration and upper-airway neural reflexes by diabetic neuropathy [78]. Various mechanisms have been proposed to explain the association between insulin resistance and OSA; some of them overlap with CVD complications. For example, sympathetic activation due to hypoxia alters the glucose metabolic cycle, which results in increased cortisol and growth hormone levels, which then can deregulate insulin sensitivity [79]. OSA was also associated with dyslipidemia in several studies due to its association with a state of hypoxia. This was theorized due to the roles of sterol regulatory element-binding protein-1 (SREBP-1) and stearoyl-coenzyme A desaturase-1 (SCD-1). However, these effects were cited due to intermittent hypoxia, and a causative effect of OSA on dyslipidemia is inconclusive [80] (Figure 4).

## 4. Current Treatment Options

Advances have been made over the years to better manage the symptoms in patients with OSA. Here, we briefly review the available treatments at present and their efficacy.

### 4.1. Continuous Positive Airway Pressure (CPAP)

CPAP is considered one of the most reliable and effective methods for treating sleep apnea. The use of the term sleep apnea started in late-20th century [9]. However, many changes were made to better accommodate the needs of patients and enhancing its effectiveness. A constant pressure is applied through a tubing system to maintain upper-airway patency during sleep [48]. Many studies have established its efficacy and importance during the course of treatment, and a longer use of CPAP has been shown to be associated with increasing severity. Ravesloot et al. in their study of mathematical function formulas to test its effectiveness reported a 33.3–48.3% reduction in the AHI index upon at least 4 hours per night of CPAP use among patients with moderate OSA [81]. Despite its benefits, the adherence rate is a major concern shared by patients and providers regarding this treatment. Studies suggests that approximately 50–60% of patients discontinue its usage within the first year of their prescription and around 15% discontinue after their very first night of usage [82]. Moreover, the adherence rates were shown to be influenced by severity, body mass index (BMI), AHI, and the oxygen desaturation index (ODI). For example, Jacobsen et al. in their retrospective study of 695 patients reported a higher adherence (89%) in patients with severe OSA as compared to 71% for moderate and 55% for mild OA, and its use was higher among patients with a higher BMI, AHI, and ODI [83]. Having said that, one study showed the importance of formulating a standard protocol, comfortable pressure settings, and offering of mask choice to patients in increasing the adherence to CPAP therapy [84]. While CPAP is widely prescribed, other airway pressure devices might be available depending on individual symptoms.

We believe effective counseling from physicians could greatly impact the rates of adherence. Thus, a proper discussion regarding sleep hygiene and the pros and cons of interrupting their therapies should be thoroughly discussed by clinicians.

### 4.2. Oral/Dental Devices

Although CPAP is still considered the gold standard when it comes to the treatment of OSA, there are other treatment strategies available if CPAP is not helping or not available to use. Oral devices, most commonly the mandibular advancement device (MAD) and tongue retaining device (TRD) are used, although tongue retainer devices are relatively older devices and are becoming obsolete alongside modern innovations in MADs. However, they are still reported to be an effective alternative treatment option for OSA [85]. MAD is the most commonly used device in patients with sleep apnea, which helps to move the mandible forward, relative to the maxilla [86]. This results in the widening of the airway, which prevents closure and obstruction during sleep. Ultimately, this helps to reduce snoring. MAD is considered as the primary treatment option for mild to moderate cases of OSA, and a secondary option in severe cases for patients having difficulties with CPAP [87]. These devices are available in various designs and can be custom-made or prefabricated. The prefabricated designs are also known as thermoplastic appliances, which are relatively cheaper and the kind that can be bought over the counter. On the other hand, custom-made designs are relatively more sophisticated and expensive because they are produced in a specialized dental laboratory and require the dental imaging of the patient to ensure a good fit [87,88]. While prefabricated devices are simple in design, custom-made designs are more intricate in nature and can consist of multiple and separate parts for the lower and upper jaw. These titratable appliances can be distinguished as middle traction and bilateral thrust devices, which differs in the way they are connected and placed in the oral cavity [87,89]. According to a recent systematic review and metanalysis, there is no clear-cut answer as to which device is superior in terms of alleviating symptoms in patients with mild to moderate OSA; however, a custom-made MAD was reported to be more superior in terms of comfort and thus a more favorable compliance when compared with a prefabricated MAD (87).

Although many people find this option more comfortable than CPAP, the use of such devices varies. Several unwanted effects of using MADs have been reported, which includes jaw pain, tenderness of denture, and hypersalivation [86]. Moreover, there is no unform guideline for the use of MADs if a patient is already suffering from a pre-existing temporomandibular disorder. However, a relatively recent clinical review reported no contraindication for the use of MADs to treat OSA with a concurrent temporomandibular disorder [90]. TRDs, on the other hand, serve a similar purpose. They appear similar to a large pacifier with a space for the tongue and a defined mandibular protrusion [91]. Similar side effects are also reported for this device. While these devices have shown to be effective, similar to CPAP, the success of this alternative option also relies on patient compliance. The compliance rate pertaining to these devices is anywhere between 30–60%, which is arguably not ideal [92,93]. Most people in these studies cited discomfort, dry mouth, hypersalivation, and other side effects mentioned above to not use it regularly. This also signifies that these devices could be used for short-term periods but are not a long-term solution for these patients. Significant changes in design and comfort are warranted if these options are to be entertained continuously. It is also important to note that those with less propensity for upper-airway collapse and low loop-gain endotypes receive the greatest benefits from oral appliance therapy and upper-airway surgery [94].

### 4.3. Surgery

Usually, surgery is an option of the last resort, or when a patient insists on choosing this option after expressing frustration and discomfort from the most common treatment options previously discussed. Uvulopalatopharyngoplasty (UPPP) is the most common surgical procedure performed on patients with OSA, where some tissues from the uvula, soft palate, and/or tonsils are resected to open the upper airways [95]. Generally, surgical indications include an AHI of higher than 15, oxyhemoglobin desaturation less than 90%, and cardiac abnormalities associated with OSA. However, it can also be elected if deemed a risk factor for motor vehicle accidents, failed compliance, or intolerance to previous therapies, and polysomnographic parameters of disease [96].

In order to select the patients who are most likely to benefit from this procedure, and to reduce unnecessary harm to patients, a staging system was created, known as the Friedman staging system. In a landmark study, Friedman et al. categorized and scored patients into three different stages based on palate position, tonsil size, and BMI [97]. Based on this scoring system, and a later modification by other scientists, stage 1 patients have a success rate of approximately 81% at present. This success rate is almost decreased by half if a patient is placed in stage 2 using this scoring system. A metanalysis by Choi et al. concluded that while stage 1 is a strong predictor of success after surgery, stage 2 is a negative predictor [98]. This signifies the importance of patient selection and a careful discussion between patients and their physicians to address the efficacy rates and risks involved in this procedure.

### 4.4. Personalization of the Treatment

Personalized medicine and person-centered care have taken center stage in medicine as of late, and the ailment of OSA is of no exception. The goal of the personalization of treatment in OSA is to carefully evaluate each patient individually, identify their risk factors, and treat them according to their symptoms, keeping in mind their needs, wishes, and values [99]. To provide truly personalized care, it is vital to obtain a detailed patient history surrounding their symptoms and factors of OSA affecting their quality of life. Historically, OSA has been treated according to the severity noted by AHI and generic symptoms [100]. However, at present, we know that OSA has a wide spectrum of symptoms, and, in fact, many patients are presented without any apparent manifestations. Therefore, it is vital to target individual sets of symptoms, which in turn would also motivate treatment adherence among patients. For example, a patient with OSA who is presented with masked cardiovascular manifestations and without daytime sleepiness or snoring at night would be less likely to adhere to CPAP, since there is no perceived immediate reward for that patient. However, someone with OSA who can visibly notice a difference in their sleepiness and snoring would be more motivated to adhere to this regimen [99]. This could be one of the reasons for the low adherence to CPAP among patients with mild OSA, as the perceived burden of using CPAP everyday could outweigh the apparent benefits. This is where patient education can play a vital role, so the patients can remain cognizant of their disease process.

Another important factor in the success of personalized medicine in OSA is actively engaging in the treatment process and self-accountability. As reported for patients with OSA and other chronic diseases, an active pedagogy using telehealth resources and applications to manage CPAP reportedly had better clinical outcomes, reduced progression in disease burden, and resulted in greater self-preventive measures [99,101,102].

The targeted and personalized therapies for OSA can be grossly categorized into two groups: anatomical and non-anatomical. CPAP, oral appliances, weight loss, positional therapy, and upper-airway surgery falls under anatomical therapy, which were previously discussed. Non-anatomical therapy is broadly classified into three groups: muscle function, loop-gain, and arousal threshold [20].

In muscle function therapy, the pharyngeal muscles are targeted since they play a vital role in the patency of the upper airway. Genioglossus is yet another important dilator muscle that can cause upper-airway collapsibility when there is a state-dependent reduction in its activity [103]. It is reported that more than 30% of patients with OSA have minimal muscle responsiveness during sleep, which could further exacerbate the symptoms and collapsibility [20]. To improve patency, hypoglossal nerve stimulation and oropharyngeal muscle training have been suggested as targeted therapies. Both of these personalized interventions have been reported to reduce AHI by more than 50% in patients suffering with OSA and a prior specific reduction in muscle activity [104]. These are categorized under personalized and targeted therapies because the success of these treatment depends on individual’s Pcrit, pharyngeal shape, and site of airway collapse [20,104]. Loop-gain therapies include oxygen supplementation and carbonic anhydrase inhibitors. Supplemental oxygen tends to reduce loop-gain and lowers the AHI in selected patients. The mechanism behind this variability for oxygen therapy is unclear at present. Acetazolamide and zonisamide are two carbonic anhydrase inhibitors that have shown promising results by decreasing loop-gains by approximately 40%, while also reducing AHI by half [105,106]. Lastly, therapies for a low arousal threshold include hypnotic agents, such as eszopiclone, zopiclone, and trazodone [20,107]. Without increasing the risk of hypoxemia, these agents can increase the threshold for arousal and reduce the AHI in patients with OSA by approximately 25% to 50% [20].

It is important to realize that these interventions are called targeted for a reason; they seem to have a variable effect on patients with OSA, and several factors can influence the effectiveness of these therapies, which are beyond the scope of this paper.

## 5. Role of Clinicians

Clinicians play a vital role not only in diagnosing OSA, but also in counseling their patients as lifestyle modifications and prescription compliance play instrumental roles in the management of this medical condition [108]. Counseling can include educating patients about their diet, sleep hygiene, exercise routine, identifying risky behavior, etc. Although OSA is a complex mixture of various intricacies, there are some modifiable risk factors that can be targeted to improve the course of the disease. The major modifiable risk factors of OSA include alcohol, smoking, sleep hygiene, and BMI [109]. For example, weight loss has been reported to decrease the severity of OSA by almost 50% in moderately obese patients with additional benefits in metabolic regulation, such as glycemic control [110]. Similarly, a fixed sleep schedule with the head as elevated and upright as possible could also help reduce symptoms. This is particularly important as sleep hygiene was reported to be indirectly related to daytime sleepiness and depressive symptoms [111]. These modifiable risk factors can also indirectly reduce subsequent cardiovascular complications if targeted early on, since many of these risk factors, such as obesity, exacerbate cardiovascular events, as discussed previously. In a nutshell, counseling should focus on targeting the modifiable risk factors and uplifting patients’ quality of life, since several studies have reported a lower quality of life among OSA patients in its most symptomatic forms [112]. The literature suggests that sleep apnea is still widely underdiagnosed [113], which signifies the key role physicians can play in terms of impacting their patients’ lives. We suspect that while searching for a correct diagnosis would help narrow the gap in epidemiological variables to devise better public health policies, it could also place an additional burden on the healthcare system, as more patients would consult their physicians and seek appropriate treatment. However, by thoroughly educating their patients about the disease, clinicians cannot only reassure their patients regarding the course of their disease, but also increase the compliant rates pertaining to their treatment. Having said that, it is difficult to predict if a certain healthcare system could handle a large influx of patients with OSA upon an increase in the effectiveness of diagnostics.

## 6. Conclusions

OSA is increasingly recognized as a prevalent medical condition affecting people globally, making it a pressing public health concern. Although advances have been made over the years, a decent patient population is still undiagnosed due to wide array of reasons, resulting in increasing morbidity and mortality rates related to OSA. This medical condition can manifest itself as several comorbidities, such as cardiovascular dysfunctions, including stroke, hypertension, coronary artery disease, metabolic disorders, chronic inflammation, etc. The most commonly prescribed treatment strategy includes CPAP and oral devices, though patient compliance is a topic of concern. Given the fact that many symptoms are manageable by lifestyle modifications, counseling and education provided by clinicians play important roles. There is a crucial need to understand the many underlying mechanisms concerning OSA and its co-manifestations to better formulate targeted therapies.

## Data Availability

Not applicable.
